# Prediction of protein secondary structure based on an improved channel attention and multiscale convolution module

**DOI:** 10.3389/fbioe.2022.901018

**Published:** 2022-07-22

**Authors:** Xin Jin, Lin Guo, Qian Jiang, Nan Wu, Shaowen Yao

**Affiliations:** ^1^ Engineering Research Center of Cyberspace, Yunnan University, Kunming, Yunnan, China; ^2^ School of Software, Yunnan University, Kunming, Yunnan, China

**Keywords:** deep learning, generative adversarial networks, channel attention, protein secondary structure prediction, neural networks, protein structure prediction

## Abstract

Prediction of the protein secondary structure is a key issue in protein science. Protein secondary structure prediction (PSSP) aims to construct a function that can map the amino acid sequence into the secondary structure so that the protein secondary structure can be obtained according to the amino acid sequence. Driven by deep learning, the prediction accuracy of the protein secondary structure has been greatly improved in recent years. To explore a new technique of PSSP, this study introduces the concept of an adversarial game into the prediction of the secondary structure, and a conditional generative adversarial network (GAN)-based prediction model is proposed. We introduce a new multiscale convolution module and an improved channel attention (ICA) module into the generator to generate the secondary structure, and then a discriminator is designed to conflict with the generator to learn the complicated features of proteins. Then, we propose a PSSP method based on the proposed multiscale convolution module and ICA module. The experimental results indicate that the conditional GAN-based protein secondary structure prediction (CGAN-PSSP) model is workable and worthy of further study because of the strong feature-learning ability of adversarial learning.

## 1 Introduction

Proteins play important roles in life activities, such as signal transduction and transmission, living material transportation, catalysis, and immunity (Saini and Hou, 2013; Pka et al., 2021). The function of a protein depends on its three-dimensional structure, which is determined by the protein sequence and folding activities within a living cell (Zou, 2000). The three-dimensional structure of a protein can be obtained by X-ray crystallography, multi-dimensional magnetic resonance, and cryo-electron microscopy, which are expensive and time-consuming, and these data are generally provided in the Protein Data Bank (PDB) (PDB, 1971; Berman and Henrick Nakamura, 2003; Kim et al., 2008). Hence, it is important for computer scientists to be able to predict the three-dimensional structures of proteins from their sequences rapidly and relatively inexpensively (Uniprot; Yang et al., 2016).

The protein secondary structure is the bridge of three-dimensional structures and sequences, which is determined by the effect of hydrogen bonds in the polypeptide chain (Rafid et al., 2020; Grmez et al., 2021; Guo et al., 2021; Sharma and Srivastava, 2021; Singh et al., 2021). Many studies have shown that we can learn the three-dimensional structures by their secondary structures, and thus the study of the protein secondary structure can improve the accuracy of three-dimensional structure prediction (Fischer and Eisenberg, 1996; Zhou and Karplus, 1999; Ozkan et al., 2007; Wu et al., 2007). Fortunately, computer software and machine learning methods can help us predict the protein secondary structure based on the protein amino acid sequence.

Since Chothla and Levitt (Levitt and Chothia, 1976) proposed the first method for protein secondary structure prediction (PSSP) in 1976, the development of PSSP has spanned three stages (Cheng et al., 2020; Zhang et al., 2020). In the first stage, the prediction accuracy of three states was about 60%–70%, such as in the methods of Chou and Fasman (1974) (50%–60%) and GOR (Garnier J, Osguthorpe DJ, Robson B) (64.4%) (Garnier et al., 1978), and most of these methods relied on the statistical probability of the individual residue that corresponds to the secondary structures. In the second stage, the neighboring residue information of the protein was considered by a sliding window, but the prediction accuracy was still less than 65%, such as in the GOR III method (Kloczkowski et al., 20022002). In the third stage, multiple sequence alignment (MSA) profiles, such as position-specific scoring matrices, were employed for PSSP (Altschul et al., 1997), and the evolutionary information helped to increase prediction accuracy to 70%, such as in PHD (Rost and Sander, 1993; Rost et al., 1994) (72.9%) and PSIPREDH (Jones, 1999) (76.5%). In the last decade, machine learning methods have been used in PSSP, including support vector machine (SVM) (Chatterjee et al., 2011), neural networks (Mirabello and Pollastri, 2013), and fuzzy set theory (Nguyen et al., 2015). Since 2015, deep-learning–based methods have been used in PSSP to improve prediction accuracy (by more than 80%), such as SPINE (Dor and Zhou, 2007) (80%), SPIDER2 (Heffernan et al., 2015) (82%), deep conditional neural fields (DeepCNF) (Wang et al., 2016a) (84%) and CRRNN (Zhang et al., 2018) (86%).

The secondary structures are impacted by the internal hydrogen bond in the polypeptide chain. Initially, researchers classified the secondary structures of a protein into only three states: helix (H), strand (E) and coil (C). Subsequently, the three states were expanded to eight states to describe proteins with more detailed local structure information (Jiang et al., 2017). Most of the earlier methods perform well in three-state prediction but perform poorly in eight-state prediction because of the increased complexity. To address this problem, many neural network–based methods have been explored for eight-state prediction, including RaptorX-SS (Wang et al., 2011) (64.8%), DCRNN (Li and Yu, 2016) and GSN (Zhou and Troyanskaya, 2014) (66.4%). Compared with conventional methods, deep learning has achieved excellent performance in feature extraction and classification, and in recent years, the prediction accuracy of eight states in PSSP has been improved by deep neural networks such as DeepCNF (Wang et al., 2016a) (68.3%), MUFOLD-SS (Fang et al., 2018) and CNNH_PSS (Zhou et al., 2018) (70.3%). In addition, the fusion of the multi-features of proteins is becoming an attractive means of improving performance, for example, the fusion of amino acid sequences and the multiple-sequence alignment profile (Wang et al., 2016a). In GSN, the protein sequence and position-specific scoring matrix (PSSM) have been combined for the prediction of eight states; in CRRNN (Zhang et al., 2018), the PSSM and physicochemical properties have been fused.

Generative adversarial networks (GANs) have achieved superior performance in feature extraction and signal reconstruction, and are widely used in image generation and classification problems. Although we can regard PSSP as a classification problem, a search of the literature did not reveal any GAN-based PSSP research to date; thus, in this study we introduce GAN into the PSSP field. GAN was proposed based on the zero-sum game theory by Goodfellow et al. (Ian et al., 2014) In the GAN, a generator and discriminator are designed to conflict with each other; the generator learns the distribution of sample data to generate fake data, and the discriminator is used to determine if its input is the ground truth or fake data produced by the generator. Through this antagonistic process, GANs have achieved outstanding performance in feature extraction and learning. GANs are widely used in image processing, signal processing, natural language processing, and biological information processing. Inspired by previous studies (Ian et al., 2014; Mehdi and Simon, 2014), we posit that GAN has a promising future in PSSP.

Leveraging the study of deep learning and PSSP, this study introduces the conditional GAN-based PSSP (CGAN-PSSP) model, in which the protein sequence and the corresponding PSSP are used as the inputs, while the secondary structure is used as the output. In this model, the secondary structure of the protein sequence is generated by the generator, and the discriminator is used to determine the authenticity of the secondary structure. After model training, the generator is used as the predictor for the protein secondary structure. We also propose a PSSP method based on our proposed multiscale convolution module and improved channel attention (ICA) module. The proposed ICA module is added to the multiscale convolution module and classification module so that the proposed model can automatically understand the importance of different functional channels. The experimental results show that our proposed model also achieves considerable performance in PSSP.

## 2 Background knowledge

This section provides background knowledge, such as input features, output features, and CGANs.

### 2.1 Input features

In this study, the one-hot form of the protein sequence is connected with the corresponding PSSM as the input features. The 20 natural amino acids are presented as A, C, D, E, F, G, H, I, K, L, M, N, P, Q, R, S, T, V, W, Y, and other unknown amino acids are denoted by X. Thus, the primary structure of any protein can be expressed by a sequence of 21 letters. To translate the protein sequence into a form that the prediction model can easily learn, each amino acid is converted into a one-hot form with a size of 1 × 21, in which there are only two elements, with values of 0 or 1, and the position of value 1 corresponds to the class of amino acid. The rest of the elements are set to 0. Thus, the protein with N amino acids will be converted into a vector with the size of N × 21. The corresponding one-hot coding forms of the 21 amino acids are described as Eq. 1:

$$\begin{array}{l} A\to \left[1,0,0,0,\cdots ,0,0\right]\\ C\to \left[0,1,0,0,\cdots ,0,0\right]\\ \;\cdots \cdots \\ X\to \left[0,0,0,0,\cdots ,0,1\right] \end{array}$$ (1)

**FIGURE 1:**
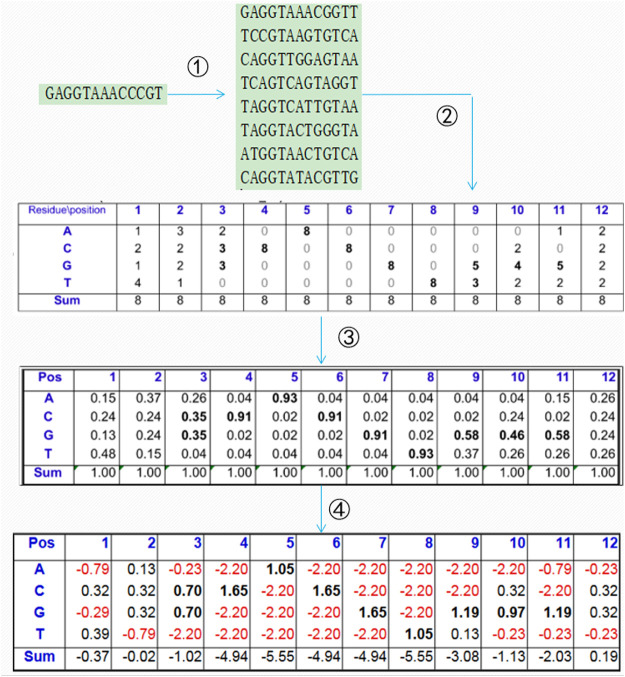
Construction process of the position-specific scoring matrix.

A PSSM is generally used to present the evolutionary information of biological sequences, and it can find a long-range correlation of the residue sequence. As shown in Figure 1, the PSI-BLAST (Altschul et al., 1997) algorithm is often used to obtain the PSSM of protein sequences according to four steps: 1) all of the sequences that are similar to the given sequence in the database are found; 2) the position frequency matrix of each amino acid is constructed; 3) the position probability matrix of each amino acid is constructed; and 4) the final PSSM is produced.

**FIGURE 2:**
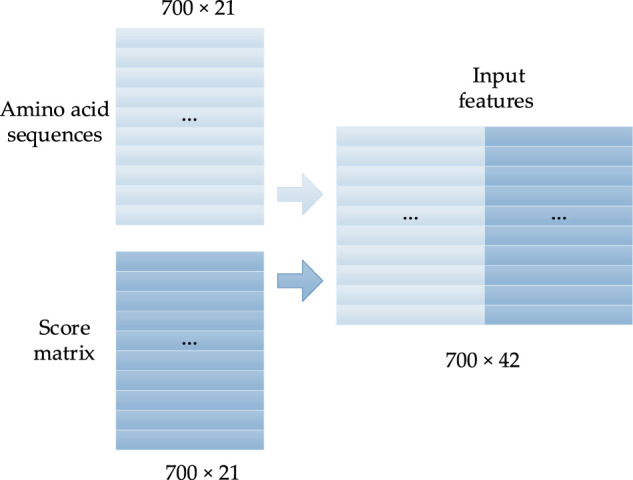
Concentrated matrix of the PSSM and one-hot form of protein sequences (Guo et al., 2020).

In this study, the size of the PSSM is N × 21, and the S-shaped function $s\left(x\right)=1/e^{-x}$ is used to normalize the scoring matrix into the range of [0, 1]. As the length of most protein sequences is less than 700, the one-hot coding of residue sequences and the size of the PSSM are generally unified into 700 × 21. That is, the sequences whose length is greater than 700 will be divided into two overlapping sequences, while the sequences whose length is less than 700 will be augmented by filling in zeros. Thus, the input feature of the prediction model is a matrix with the size of 700 × 42, as shown in Figure 2. In the constructed matrix, the first to 21st columns are the one-hot coding form of the residue sequence, and the 22nd to 42nd columns in each row are the PSSM of the corresponding amino acids.

### 2.2 Conditional GANs

**FIGURE 3:**
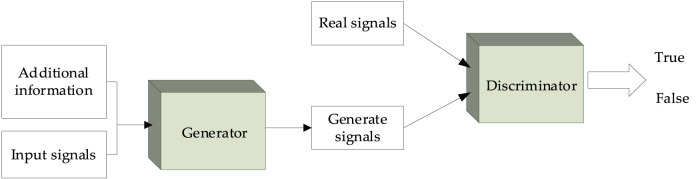
Block diagram of the CGAN.

The GAN was proposed in 2014 by Goodfellow et al. (Ian et al., 2014) based on the zero-sum game theory. A GAN usually consists of a generator and a discriminator, which can improve the performance of the generator in adversarial learning. Also in 2014, Mehdi and Simon (2014) proposed a CGAN by adding conditional information. The main idea of the CGAN is to add relevant conditional information to the generator and discriminator, enabling the model to conditionally generate specific signals. The overall structure of the CGAN is shown in Figure 3.

In the generator, a given input signal and additional information are used as the input of the neural network to output the generated signal. Then, the discriminator determines whether its input signal is true or false. In this work, the generator is used to generate the “false” secondary structure, and the discriminant is used to judge the authenticity of the secondary structure. When the input signal of the discriminant is the secondary structure generated by the generator, the discriminant should discriminate it as “false”; when the real secondary structure is inputted into the discriminant, it should be discriminated as “true.” The loss function is then used to calculate the errors in the judgment of the discriminator. Thus, the generator and discriminator conflict with each other.

In the CGAN, it is expected that the generator can generate false signals that infinitely approach the real signals; it is also expected that the discriminator can accurately distinguish the true and false signals under the given conditions. Hence, the loss function of the CGAN is constructed as follows:

$$\underset{G}{\min}\underset{D}{\max}V\left(D,G\right)=E_{x∼p_{data}}\left[\log D\left(x|y\right)\right]+E_{z∼p_{z}\left(z\right)}\left[\log\left(1-d\left(G\left(x|y\right)\right)\right)\right],$$ (2)

where p _z_ (z) is the input signal, G is the generator, D is the discriminator, and P_data_ represents the real data.

## 3 Proposed CGAN-PSSP model

In this study, we propose a novel PSSP based on the CGAN, called CGAN-PSSP, which is described below.

### 3.1 Overview of the proposed CGAN-PSSP model

**FIGURE 4:**
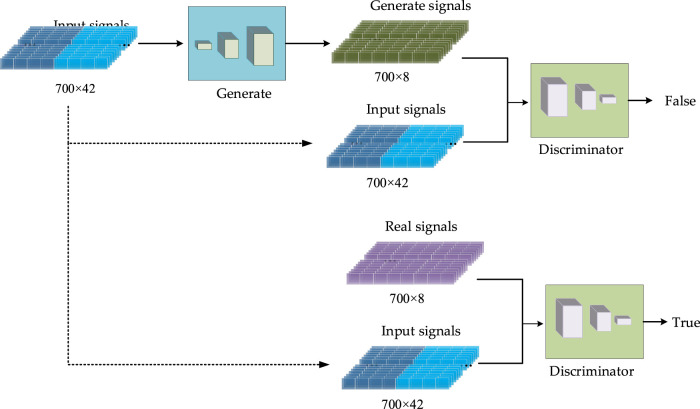
Schematic of the proposed CGAN-PSSP.

The proposed CGAN-PSSP has a generator and a discriminator. In this model, the input of the generator is a 700 × 42 vector composed of amino acid coding features and a PSSM, and the output is a 700 × 8 (eight-state) or 700 × 3 (three-state) vector that is the predicted protein secondary structure. Thus, the generator is the predictor behind the Protein secondary structure prediction. The input of the discriminator is the combination of the secondary structure and the input feature of the generator, and the output is the discriminant results, as shown in Figure 4. When the secondary structure is real, the result of the discriminant should be true; otherwise, the generated result of the generator should be determined as false. For the generator, we expect the secondary structure to be as realistic as possible; for the discriminator, it is expected to always determine that the secondary structure generated by the generator is false. In the end, we expect a balance should be reached in the game. Because the purpose of CGAN-PSSP is to construct a powerful generator, the structure of the generator should be slightly more complex to generate a sufficiently realistic “false secondary structure.” The main flow of the CGAN-PSSP model is shown in Figure 4.

### 3.2 Generator

**FIGURE 5:**
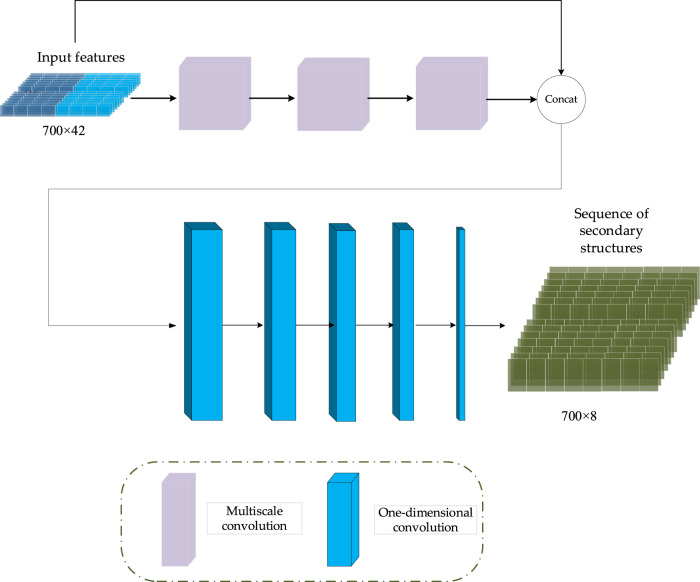
Generator structure of CGAN-PSSP.

In CGAN-PSSP, the key function of the generator is to generate a “false” sequence of secondary structures based on the input features of protein sequences. The generator of CGAN-PSSP combines one-dimensional convolution (Guo et al., 2020), and our proposed multiscale convolution to capture the complex features of proteins. The one-hot form of the protein sequence and PSSM are combined as the input feature of the generator. Three continuous multiscale convolutions are used to extract the features, and the 700 × 42 input feature is upsampled to 700 × 2048. To prevent the loss of the original feature, the input feature with the size of 700 × 42 is connected to the output of the multiscale convolution module, and a feature map with the size of 700 × 2090 is then produced. Subsequently, a one-dimensional convolution module is used to subsample the 700 × 2090 feature map into a 700 × 8 or 700 × 3 feature map that corresponds to the eight states and three states of the secondary structure prediction. The structure of the generator is shown in Figure 5, and the hyperparameters are shown in Table 1.

**TABLE 1:** Hyperparameters of the generator structure in CGAN-PSSP.

Operation	Input	Convolution kernel size	Step	Output
Multiscale convolution	700 × 42	11	1	700 × 256
Multiscale convolution	700 × 256	11	1	700 × 512
Multiscale convolution	700 × 512	11	1	700 × 2048
Concatenation	700 × 2048	—	—	700 × 2090
	700 × 42			
One-dimensional convolution	700 × 2090	11	1	700 × 512
One-dimensional convolution	700 × 1,024	11	1	700 × 128
One-dimensional convolution	700 × 512	11	1	700 × 32
One-dimensional convolution	700 × 128	11	1	700 × 16
One-dimensional convolution	700 × 64	11	1	700 × 8 (700 × 3)

#### 3.2.1 Multiscale convolution module

**FIGURE 6:**
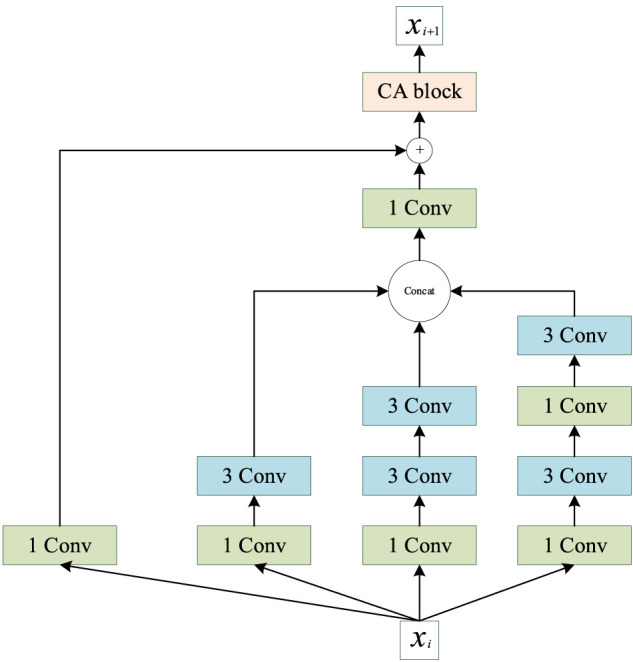
Schematic of the proposed multiscale convolution (MSC) module.

Inception (Szegedy et al., 2015) was the first concept of multiscale convolution, and several modified versions have since been proposed to improve the performance (Szegedy et al., 2016; Szegedy et al., 2017). Inspired by the Inception network, we introduce an improved multiscale convolution (MSC) module into PSSP to extract the features of protein sequences. As shown in Figure 6, the MSC module is composed of a one-dimensional convolution operation with a convolution kernel size of 1 (1 Conv) and a one-dimensional convolution operation with a convolution kernel size of 3 (3 × 3 Conv). The Mish function (Misra, 1908) is used as a nonlinear activator. Moreover, an ICA module is used in the MSC module to obtain the importance of each channel. In the proposed MSC module, Xi represents the input of layer i, Xi + 1 represents the output of layer i, and the ICA block represents the ICA module.

#### 3.2.2 Improved channel attention module

The main function of the ICA mechanism is to enable the model to automatically understand the importance of each functional channel in the feature map, in order to improve the expression ability and function fitting ability of the model. The Squeeze and Excitation (SE) Net (Jie et al., 2017) is a classic ICA mechanism network that is composed of SE operations. The SE will produce a weight for each feature map of the channel to indicate the relevance between the channel and the key information.

**FIGURE 7:**
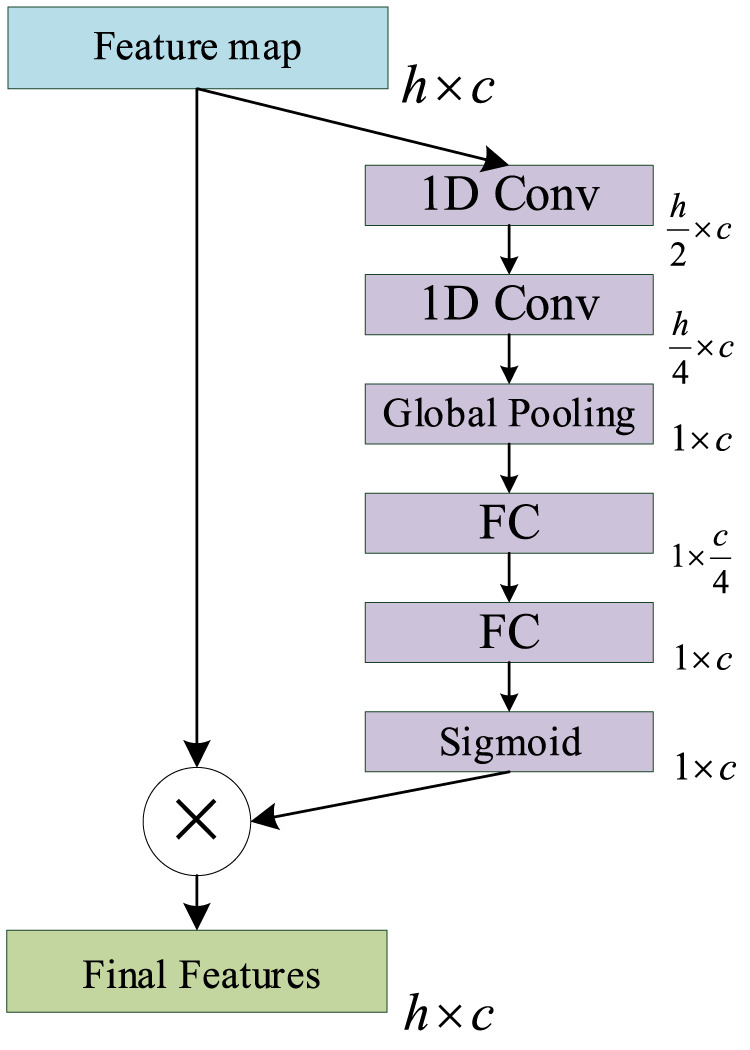
Schematic of the improved channel attention (ICA) module.

However, the number of parameters contained in the original SE Net is too small to accurately represent the importance of each channel in PSSP. Accordingly, we improved the original SE Net by adding two convolution operations to the Squeeze operation to increase the number of parameters. This allows us to improve the ability of the ICA mechanism to express the importance of each channel in PSSP. The ICA module is shown in Figure 7, in which 1D Conv represents a one-dimensional convolution operation with a convolution kernel size of 3, Global Pooling represents a global average pooling operation, and FC represents a full connection operation. The sigmoid represents the sigmoid function, and the final feature represents the feature map with channel importance. The ICA module is added to the MSC and classification modules so that the proposed model can automatically understand the importance of different functional channels.

#### 3.2.3 One-dimensional convolution module

The core ideas of the convolutional neural network (CNN) (Lecun, 1989) are the perceptual field and weight sharing. The perceptual field is used to extract the local features of input signals. The perceptual field is conducted by a convolution operation that can be regarded as a sliding window, and its mathematical formula can be described as follows:

$$y\left(k\right)=h\left(k\right)*u\left(k\right)=\sum_{i=1}^{N}h\left(k-i\right)u\left(i\right),$$ (3)

where h represents the signals, u represents the in-process signals, N is the size of the input signal, and y is the convoluted signal.

**FIGURE 8:**
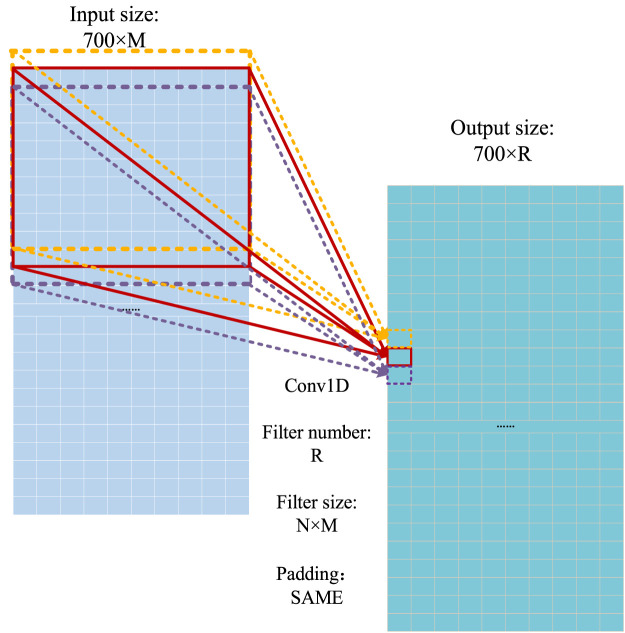
Operational process of the one-dimensional convolution (Guo et al., 2020).

The one-dimensional convolution operation (Guo et al., 2020) is used as the basic operation to extract the features of proteins. The operation process of the one-dimensional convolution in the model is shown in Figure 8, where the convoluted signal is a 700 × M matrix, and the filter size of the convoluted signal is N × M. Because the output size depends on the number of convolution signals (R), the size of the output signal is 700 × R.

### 3.3 Discriminator

**FIGURE 9:**
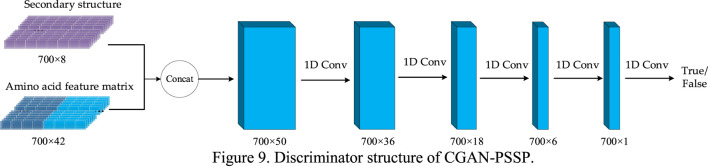
Discriminator structure of CGAN-PSSP.

In the CGAN-PSSP model, the function of the discriminator is to judge the truth or falsehood of the secondary structure. If the secondary structure generated by the generator is false, the judgment of the discriminator should be false. For the real secondary structure sequence, the judgment of the discriminator should be true. Figure 9 depicts the structure of the discriminant, whose input is a combination of the secondary structure and amino acid feature matrix. Therefore, when the model is used to predict three states, the size of the input feature is 700 × 45; when the model is used to predict eight states, the size of the input feature is 700 × 50.

Four continuous one-dimensional convolutions are used to sample the input features into a map with the size of 700 × 1. Finally, the sigmoid function is used to convert all the values of the output matrix into the probability of [0, 1]. Each value in the output matrix represents the truth or falsehood of the corresponding residue on the secondary structure sequence. In the training process, when the secondary structure is true, the output is a matrix with all values of 1. When the secondary structure is false, the output is a matrix whose values are all 0. In the testing process, if the value in the output matrix is greater than 0.5, the corresponding secondary structure is judged to be true; if the value is less than or equal to 0.5, it is judged to be false. Table 2 lists the parameter settings of the discriminator in the CGAN-PSSP model.

**TABLE 2:** Hyperparameters of discriminator structure on eight-state prediction.

Operation	Input	Convolution kernel size	Step	Output
Concatenation	700 × 42, 700 × 8	—	—	700 × 50
One-dimensional convolution	700 × 50	3	1	700 × 36
One-dimensional convolution	700 × 36	3	1	700 × 18
One-dimensional convolution	700 × 18	3	1	700 × 6
One-dimensional convolution	700 × 6	3	1	700 × 1
Sigmoid	700 × 1	—	—	700 × 1

### 3.4 Loss function

In this work, the discriminator uses the mean square error (MSE) function as the loss function. Cross-entropy is a popular loss function in deep learning for classification problems (Bahri et al., 2021; Zhu et al., 2021). To prevent the prediction model from becoming overfitted with the increase of the weight, this study introduces an improved version of the cross-entropy function to improve the performance according to the characteristics of the secondary structure, so that the performance is satisfactory for one-hot distribution as well as uniform distribution of data. The improved loss function formula is

$$loss=\left(1-\varepsilon \right)\left[-\sum_{x}p\left(x\right)\log\left(q\left(x\right)\right)\right]+\varepsilon \overset{n}{\underset{i=1}{\sum }}\frac{1}{n}\left[-\sum_{x}\log\left(q\left(x\right)\right)\right],$$ (4)

where ε∈(0,1), x represents the label category, p(x) represents the probability distribution of the true value when the label category is x , q(x) represents the probability distribution of the predicted value when the label category is x , $\varepsilon \in \left(\text{0,}1\right),\text{E}=\sum_{i=1}^{n}e_{i}$ , and n represents the number of the label categories.

## 4 Proposed MCNN-PSSP model

We propose a multiscale CNN to the secondary structure, called MCNN-PSSP, based on the MSC module and ICA module.

### 4.1 Overview of the proposed CGAN-PSSP model

**FIGURE 10:**
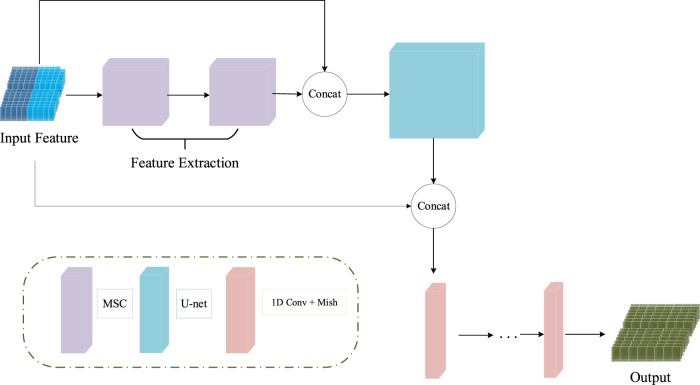
Schematic of the structure of MCNN-PSSP. Concat, concatenation operation; MSC, multiscale convolution module; 1D Conv, one-dimensional convolution operation.

The input features of the MCNN-PSSP model are the combination of protein-coding features and the PSSM. First, the MSC module expands the size of 700 × 42 input sequence features to 700 × 256 in order to extract original features. To prevent feature loss, the 700 × 42 input feature is connected to the output of the MSC module. Then, the classification module convolves the 700 × 298 feature tensor into the output tensors with a size of 700 × 8 or 700 × 3, which corresponds to the eight or three states of the protein secondary structure, respectively. The ICA module is added to the MSC module and the classification module so that the model can automatically understand the importance of different functional channels. The overall framework of the model is shown in Figure 10. The parameter settings are listed in Table 3.

**TABLE 3:** Hyperparameters of MCNN-PSSP

Operation	Input	Convolution kernel size	Step	Output
Multiscale convolution	700 × 42	11	1	700 × 84
Multiscale convolution	700 × 168	11	1	700 × 256
Concatenation	700 × 256			700 × 298
	700 × 42			
U-net	700 × 298	11	1	700 × 298
Concatenation	700 × 298			700 × 340
	700 × 42			
One-dimensional convolution	700 × 340	11	1	700 × 210
One-dimensional convolution	700 × 210	11	1	700 × 128
One-dimensional convolution	700 × 128	11	1	700 × 64
One-dimensional convolution	700 × 64	11	1	700 × 32
One-dimensional convolution	700 × 32	11	1	700 × 16
One-dimensional convolution	700 × 16	11	1	700 × 8 (700 × 3)

### 4.2 Prediction module

**FIGURE 11:**
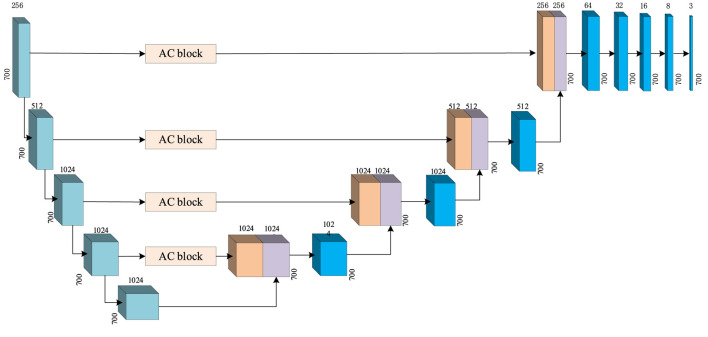
Prediction module in MCNN-PSSP.

The classification module in MCNN-PSSP is used to analyze the extracted features and carry out the secondary structure classification of three or eight states. The structure of the classification module is depicted in Figure 11, which is composed of a U-net and a one-dimensional convolution. U-net consists of a downsampling process and upsampling process, which are symmetrical in structure. Skipping connections can enhance the contact between the shrinking path and expanding path. To better analyze the extracted features, the ICA module is also integrated into the U-net. The one-dimensional convolution module consists of one-dimensional convolution operations, batch regularization, and Mish activation functions. It is responsible for converting a 700 × 512 feature map into a 700 × 8 or 700 × 3 output, which corresponds to the eight or three states of the secondary structure, respectively.

## 5 Experiment and analysis

To verify the performance of the proposed model, we used several popular datasets and metrics in this work.

### 5.1 Index

The Q score (Jiang et al., 2017) was used to evaluate the performance of the proposed PSSP model. Q score is defined as the percentage of residues that is correctly predicted in all amino acid residues, and its formula can be expressed as

$$Q_{c}=\frac{1}{res}\sum_{i=1}^{c}T_{ii},$$ (5)

where c is the number of labels, the three states correspond to Q3, the eight states correspond to Q8, res is the number of all amino acid sequences, and *T*_*ii*_ represents the correct number of amino acids in the *i*-state.

### 5.2 Datasets

Four datasets were used in this study:

1) CB513 (Cuff and Barton, 1999) was proposed by Cuff and Barton, and the similarity among these proteins is less than 25% to ensure minimal homology. Therefore, it is a dataset that contains no homologous proteins.
2) CullPDB (Wang and Dunbrack, 2003) was produced with PISCES CullPDB server; it is a large dataset containing no homologous proteins, and each residue sequence has a corresponding secondary structure. Because CullPDB and CB513 have redundant information on the sequences, the sequences whose similarity in CULLPDB is greater than 25% to those in CB513 are deleted, and many repeat sequences in CullPDB are also discarded. Thus, only 5,365 protein sequences remain.
3) CASP (Predictioncenter) is a non-homologous protein dataset constructed for a biennial protein structure prediction competition. To compare the proposed method with other prediction models, CASP10 (Kryshtafovych et al., 2014) and CASP11 (Moult et al., 2014) are employed, which have the same characteristics. These two datasets contain 123 sequences and 105 sequences, respectively, and are the most frequently used datasets in recent years.

The above datasets are publicly available and can be accessed from the relevant websites. CullPDB and CB513 are provided at http://www.princeton.edu/∼jzthree/datasets/ICML2014/. CASP10 and CASP11 can be downloaded from http://predictioncenter.org/. In keeping with other prediction models, these four datasets were preprocessed as follows: CullPDB was split into three subsets with sequences 1–4,850 used only for training, sequences 4,850–5,053 used only for verification, and the remaining 272 used for testing, and the remaining three datasets were only used for testing the model.

### 5.3 Model training

The CGAN-PSSP model was trained on the Nvidia’s Titan RTX GPU. The model structure was implemented by Keras, and the Mish and Softmax functions were used as activators for the model. The weight was initialized by MSRA, and Adam optimization algorithm (Kingma and Ba, 2014) was used to automatically update the weight and learning rate of the model. The training time was set to 750, because the prediction accuracy of the model tended to be stable. Table 4 shows the Q8/Q3 training accuracy and validation accuracy of the proposed methods on the CullPDB dataset.

**TABLE 4:** Q8/Q3 of the proposed methods on CullPDB.

	Training set (%)	Validation set (%)	Training set (%)	Validation set (%)
Q8 accuracy	86.7	75.1	87.4	84.1
Q3 accuracy	92.4	85.9	96.5	87.2
	CGAN-PSSP	CGAN-PSSP	MCNN-PSSP	MCNN-PSSP

### 5.4 Model testing and comparison

The remaining 272 sequences in CullPD, SB 513, CASP10, and CASP11 were only used for testing the model. Tables 5, 6 show the Q8 accuracy and Q3 accuracy of CGAN-PSSP and other prediction methods, respectively, on the four testing sets. The Q8 accuracy and Q3 accuracy of the CGAN-PSSP model on the four test sets show the CGAN-PSSP model is not competitive when compared with the accuracy of the other models. However, MCNN-PSSP is more competitive than the other methods in terms of Q8 and Q3 accuracy. The prediction model of CGAN-SS is different from the current deep-learning-based method, and it is an adversarial learning model. In our method, a generator and discriminator are designed to conflict with each other. The generator learns the distribution of sample data to generate fake data, and the discriminator is used to determine if its input is the ground truth or fake data produced by the generator. Thus, the GAN-based method can reduce the dependence of the training dataset in the PSSP. However, the performance of other deep-learning–based methods relies on the training datasets, which are difficult to acquire and limited in quantity. For a given dataset, in terms of the Q8 accuracy and Q3 accuracy, the CGAN-PSSP is not competitive compared with the other models. Because this is an exploratory work to verify the performance of the GAN for PSSP, we show that the feature learning and pattern classification ability of adversarial learning is workable in this field. However, we acknowledge that there is a good deal of room for performance improvement in GAN-based PSSP.

**TABLE 5:** Q8 of different prediction models (-- means no testing).

Method	CullPDB (%)	CB513 (%)	CASP10 (%)	CASP11 (%)
RaptorX-SS Wang et al. (2011)	69.7	64.9	64.8	65.1
GSN Zhou and Troyanskaya (2014)	72.1	66.4	—	—
DeepCNF Wang et al. (2016a)	75.2	68.3	71.8	72.3
DCRNN Li and Yu (2016)	--	70.4	73.9	71.2
SSREDN Wang et al. (2016b)	73.1	68.2	—	—
CNNH_PSS Zhou et al. (2018)	74.0	70.3	—	—
MUFOLD-SS Fang et al. (2018)	—	70.5	74.2	71.6
CRRNN Zhang et al. (2018)	—	71.4	73.8	71.6
F1DCNN-SS Guo et al. (2020)	74.1	70.5	74.9	71.3
MCNN- PSSP	74.2	70.6	74.9	71.5
CGAN- PSSP	74.0	70.3	74.6	71.3

**TABLE 6:** Q3 of different prediction models (-- means no testing).

Method	CullPDB (%)	CB513 (%)	CASP10 (%)	CASP11 (%)
RaptorX-SS Wang et al. (2011)	81.5	78.3	78.9	79.1
JPRED Cuff et al. (1998)	82.5	83.3	82.4	82.0
DeepCNF Wang et al. (2016a)	85.4	82.3	84.4	84.7
SSREDN Wang et al. (2016b)	84.2	82.9	—	—
MUFOLD-SS Fang et al. (2018)	—	82.7	84.3	82.3
CRRNN Zhang et al. (2018)	—	85.3	86.1	84.2
F1DCNN-SS Guo et al. (2020)	86.2	84.5	87.8	84.7
MCNN-PSSP	86.3	84.7	87.7	84.8
CGAN-PSSP	86.0	84.3	87.4	84.8

## 6 Conclusion

In this work, we proposed CGAN-PSSP, a novel PSSP model based on CGAN, which can be used to predict the eight-state and three-state protein secondary structure. In the proposed model, the generator is used to predict the secondary structure of proteins with the input of the PSSM and protein sequences, and a discriminator is designed to conflict with the generator. Accordingly, the generator can learn the complicated features of protein sequences to predict the protein secondary structure. In addition, we introduce a new multiscale convolution that has a modified ICA module. This study demonstrates that GAN can be used for PSSP, and that generative adversarial learning has great potential for protein structure prediction. Furthermore, we combined U-net with the proposed MSC and ICA modules to propose a PSSP method. However, improvements can be made in several areas, such as in the loss function and model structure. The experimental results indicated that the proposed methods achieved satisfactory performance compared with other conventional models and that the proposed multiscale convolution module and ICA module were effective.

GAN is a neural network model based on zero-sum game theory. In GAN, a generator and discriminator are designed to conflict with each other, the generator learns the distribution of sample data to generate fake data, and the discriminator is used to determine if its input is the ground truth or fake data that are produced by the generator. Through this antagonistic process, GAN has outstanding capability in feature extraction and learning compared with conventional model structures. The structure of GAN can have a strong influence on the performance of PSSP tasks; however, questions about model structure construction, model training, and loss function remain to be answered. Furthermore, the proven structures and modules of GAN in image generation tasks are worthy of study in PSSP tasks because of their superior performance in feature extraction and signal reconstruction.

## Data Availability

The original contributions presented in the study are included in the article/supplementary material, further inquiries can be directed to the corresponding author.
